# Effects on Posture of a Two-Diopter Horizontal Prism Base Out on the Non-Dominant Eye

**DOI:** 10.3390/jcm13247847

**Published:** 2024-12-23

**Authors:** Davide Marini, Giovanni Rubegni, Lorenzo Sarti, Alessandra Rufa, Marco Mandalà, Fabio Ferretti, Gian Marco Tosi, Mario Fruschelli

**Affiliations:** 1 Ophthalmology Unit, Department of Medicine, Surgery and Neuroscience, University of Siena, 53100 Siena, Italy; 2 School of Orthoptic and Ophthalmologic Assistance, Department of Medicine, Surgery and Neuroscience, University of Siena, 53100 Siena, Italy; lorenzosartiortottista@gmail.com; 3 Eye Tracking & Visual Application Lab (EVALab), Neurology and Neurometabolic Unit, Department of Medicine, Surgery and Neuroscience, University of Siena, 53100 Siena, Italy; 4 Otolaryngology Unit, Department of Medicine, Surgery and Neuroscience, University of Siena, 53100 Siena, Italy; 5 Department of Medicine, Surgery and Neuroscience, University of Siena, 53100 Siena, Italy

**Keywords:** horizontal phoria, prism, heterophoria, vergence, ocular proprioception, posture, balance control, posturography, EquiTest NeuroCom, sensory organization test

## Abstract

**Background/Objectives**: Ocular proprioception is implicated in balance control and heterophoria is associated with abnormal posture, though previous research focused mainly on the role of vertical phoria and the use of vertical prisms. This study aims to evaluate whether ocular misalignment and prismatic correction of horizontal phoria affect posture. **Methods**: Sixty-nine (*N* = 69) young healthy subjects were included and equally divided by horizontal distance phoria: orthophoria (*n* = 23), esophoria (*n* = 23) and exophoria (*n* = 23). A prism of low power (two-diopter) was placed base out on the non-dominant eye, reducing misalignment in esophorics and increasing it in exophorics more than in orthophorics. Dynamic computerized posturography was performed with the sensory organization test protocol (SOT) of the EquiTest^®^ NeuroCom^®^ version 8 platform both without and with prism, always maintaining subjects unaware of prism use. A mixed model for repeated measures analysis of variance was run to evaluate the main effect of prism and the interaction effect of prism with baseline phoria. **Results**: Composite movement strategy score without prism was 88.1 ± 2.8% (ankle-dominant strategy) and slightly increased to 89.0 ± 3.1% with prism insertion (*p* = 0.004), further shifting toward ankle strategy. Composite equilibrium score without prism was 80.3 ± 6.5% and remained stable with prism insertion (81.3 ± 8.2%, *p* = 0.117), medio-lateral and antero-posterior projection of center of gravity did not displace significantly under prism insertion (*p* = 0.652 and *p* = 0.270, respectively). At baseline, posturographic parameters were statistically independent of individual phoria, and no significant interaction between prism insertion and individual phoria was documented for any parameters (*p* > 0.05 for all). Secondary analysis and pairwise comparisons confirmed that the effect of prism was strongly selective on condition SOT 5 (eyes-closed, platform sway-referenced) with improvement of equilibrium (70.4 ± 9.7% with prism vs. 65.7 ± 11.6% without) and more use of ankle strategy (81.6 ± 5.3% with prism vs. 78.2 ± 6.0% without), without any interaction of phoria and ocular dominance, while the other conditions were comparable with and without prism. **Conclusions**: A two-diopter prism base out on the non-dominant eye induces the body to use the ankle joint more independently of individual phoria, suggesting a small improvement in postural control, while maintaining oscillations of the center of gravity unaltered. Prism seems to enhance the function of vestibular system selectively. Phoria adjustments with prismatic correction enable intervention in postural behavior. Extraocular muscles could act as proprioceptors influencing postural stability.

## 1. Introduction

Phoria is the tendency of both eyes to deviate from the parallel when fusion is blocked (latent deviation), in contrast to tropia, which is a misalignment under binocular vision (manifest deviation) [1].

Postural stability is the ability to control the body’s center of mass in relation to a person’s base of support [2] and is maintained by a close interconnection between the somatic (cutaneous), proprioceptive (joint), vestibular and visual systems [3,4].

Both eyes participate in postural control through binocular vision and stereopsis [5], although a fascinating hypothesis considers the role of extraocular muscles (EOMs) as afferent sensory receptors (proprioceptors) influencing efferent pathways of balance (postural receptors). Extraocular muscles are richly innervated by sensory receptors of the trigeminal nerve forming a proprioceptive system [6,7], which is mainly implicated in the coordination of extraocular movements and spatial coding [8,9]. Some clinical studies on vertical phoria raised the possibility that eyes act as postural receptors as well [10,11,12]. In this way, heterophoria may stimulate the ocular proprioceptive system through changes in EOMs activity (efference copy—outflow model) [6,13,14,15,16] and tension (re-afferent signal—inflow model) [6,16,17,18], thereby influencing postural control [19]. Both these signals are involved in several motor and sensory pathways as well: awareness of eye position [20], sensory interpretation of binocular correspondence to avoid diplopia [9], long-term maintenance of ocular alignment (phoria) and coding of visual target position and velocity with respect to the head [8].

Small vertical prisms are already known to induce changes in the postural behavior of healthy subjects [21,22,23,24], and prismatic correction of vertical heterophoria may improve balance performances [10]. However, little is known about the role of horizontal phoria and prisms on balance control: only a single study explored the effects of vergence-inducing horizontal prisms [25], although neglecting the influence of horizontal phoria and evaluating only general postural instability.

Therefore, the purpose of this study is to evaluate the effects of both horizontal phoria and prisms on postural control and balance maintenance strategy in healthy individuals. These aspects can be easily quantified by computerized posturography through the NeuroCom^®^ EquiTest^®^ sensory organization test (SOT) platform, which measures the subject’s ability to maintain equilibrium (body sway) and the movement strategy used to keep balance (torque of hip and ankle joints) in upright stance under different sensorial conditions (somatic, visual and vestibular) [26].

## 2. Materials and Methods

This study respected the Declaration of Helsinki ethical standards and detailed written informed consents explaining the experimental procedures were obtained prior to participation. Ethical review and approval were waived for this study due to the observational and noninvasive nature of the study.

### 2.1. Subjects Selection

Subjects were randomly recruited from the University of Siena Medicine Faculty register after careful medical history, ophthalmic and orthoptic evaluations. Included subjects were also asked to complete a self-reported questionnaire exploring the presence of symptoms of asthenopia, which can be found in the Supplementary Materials (Section S6.1 and Table S2).

The inclusion criteria were being aged between 18 and 35 years, either emmetropia or ametropia, and either orthophoria or horizontal heterophoria. The exclusion criteria were spherical equivalent below −4 diopter (D) or above +2 D, respectively; astigmatism more than 1 D; monocular best-corrected visual acuity less than the 20/20 Snellen line in either eye; horizontal phoria more than 12 diopter (Δ) or any vertical phoria or cyclophoria; stereoscopic acuity worse than 100 arc-second; any systemic, ocular, vestibular or neurological disorders potentially altering posturographic performance (e.g., strabismus, amblyopia, asthenopia, diplopia, blurring, dizziness, head injury, vestibular neuritis, multiple sclerosis, spondylitis, neck/back pain, headache/migraine, motion sickness, habitual use of orthodontic bite, etc.).

Eligible subjects were assigned to one of three study groups according to horizontal distance phoria and manifest refraction: orthophoria with emmetropia, esophoria, and exophoria.

### 2.2. Orthoptics Assessment

Stereoacuity was measured in arc seconds by the Titmus test [1] and converted to the base-10 logarithm of arc seconds for statistical analysis. Ocular dominance was determined by a hole-in-card test [27]. Horizontal distance phoria was measured by a rotating Risley–Maddox prism [28] and a six-diopter prism placed base down in front of the non-dominant eye to induce dissociation of the two eyes. Exophoria was defined as alignment produced with a base-in prism stronger than 0.5 diopter, esophoria with a base-out prism stronger than 0.5 diopter, and orthophoria with a base-in or base-out prism less than 0.5 diopter.

### 2.3. Posturographic Assessment

Posturography was performed without prism and with a monocular two-diopter prism placed base out in front of the non-dominant eye. To avoid learning or emotional errors, subjects were allowed to rest between each measurement, all were unaware of prism insertion using a neutral lens to sham the prism at baseline, and the experiments with and without prism were performed for first or second at random. The trial frame setup can be found in the Supplementary Materials (Section S5 and Figure S3). During study procedures, no subject reported diplopia, eye strain or any other visual, vestibular or somatic symptoms of concern.

Static and dynamic computerized posturography was carried out with the NeuroCom^®^ EquiTest^®^ System version 8 platform (NeuroCom^®^ International Inc., Clackamas, OR, USA), selecting SOT as the standard protocol of assessment [26].

This protocol evaluates the interaction between the somatosensory, visual and vestibular systems contributing to postural control, recording the oscillations of center of gravity (COG) and the ability to select appropriate movement strategies [26] under six different sensorial conditions. The subject is placed on a dual force plate and surrounded by a panorama reproducing an open space, both oscillating in exact synchrony with each other, wearing a safety harness. The examination is composed of three trials for each of the six conditions changing for support plate inputs (fixed and sway-referenced) and visual inputs (fixed, eyes closed and sway-referenced). The system calculates four parameters from direct measurements of vertical and shear forces exerted by the subject on the force plate.

The *composite equilibrium score* (CES) quantifies the global performance. It is given by the weighted average of the maximum antero-posterior body oscillation (COG sway) across all the trials: the closer it gets to 100, the smaller the area of oscillation, a score of zero corresponding to maximal sway of 12.5° [26].

The *composite movement strategy score* (CMS) highlights the weighted average of candidate’s rotation of the ankles relative to the hips. Hip movements generate horizontal shear forces proportional to the angular acceleration of hip joint: a score of 100 highlights an adaptive strategy in total favor of the ankles (corresponding to a maximal shear force of 25 lbs [11.3 kg]), while a score of zero in total favor of the hips [26].

The *COG alignment on* *x*-axis (Px) and *y*-axis (Py), respectively, represent the medio-lateral (ML) and antero-posterior (AP) projections of the subject center of mass on the support surface measured in inches: right and forward displacements are positive, while left and backward negative.

Specific details on mechanics and computation details of the NeuroCom^®^ EquiTest^®^ platform can be found in the Supplementary Materials (Sections S1–S4).

### 2.4. Statistical Analysis

SPSS software version 26 (IBM, Armonk, NY, USA) was used for statistical analysis, and charts were plotted with Prism software version 10 (GraphPad, San Diego, CA, USA).

The main effect of prism on each posturographic parameter and its interaction with phoria were evaluated with a mixed model for repeated measures analysis of variance (ANOVA) assuming the phoria group as between-subjects factor and the prism as within-subjects factor. Baseline posturographic parameters (without prism) were analyzed between phoria groups by a one-way ANOVA, and dichotomous variables by Fisher’s exact test. Changes in distance horizontal phoria induced by prism were compared with a Wilcoxon signed-rank test in each group separately and stereoacuity changes were analyzed with a two-way ANOVA (phoria group as between-subjects factor and prism as within-subjects factor).

A total sample size of 69 subjects (23 in each group) was determined to attain a statistical power of 95%, setting type I error at 5% and assuming a moderate effect size of 5% for the main effect of prism on posturographic scores.

A secondary analysis was carried out to determine whether the effect of prism on posturographic scores depended on which eye was placed (ocular dominance) and to ascertain whether the effect of prism was different in certain SOT conditions: a repeated measures ANOVA was run including phoria and ocular dominance as between-subjects factors and prism and condition as within-subjects factors.

## 3. Results

### 3.1. Distance Phoria and Stereoacuity

**Figure 1:**
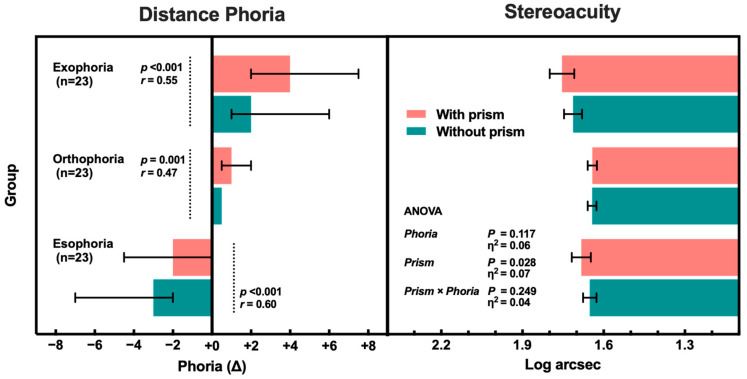
Distributions of horizontal distance phoria (Δ, prismatic diopter) and stereoacuity (log arcsec) *without* and *with prism*. Column and bar respectively represent median and interquartile range of phoria, and mean and standard error of stereoacuity. Wilcoxon signed-rank test statistical significance (*P*) and effect size (*r*) showed a significant exophoric shift in all groups after prism insertion. Two-way ANOVA statistical significance (*P*) and partial effect size (η^2^) of main effect (*Prism*) and interaction (*Prism × Phoria*) showed a significant small increase in logarithm of stereoacuity seconds of arc for all groups.

The distribution of absolute distance phoria changed after prism insertion: orthophoric subjects reduced from 33% without prism (23/69) to 9% with prism (6/69) and median absolute distance phoria changed from 1.00 [IQR 0.50, 3.75] Δ without prism to 2.00 [IQR 1.00, 4.00] Δ with prism (Figure 1).

With prism insertion, distance phoria significantly increased in orthophoric subjects (*Z* = 3.218, *p* = 0.001 and *r* = 0.47), significantly decreased in esophorics (*Z* = −4.051, *p* < 0.001 and *r* = 0.60) and significantly increased in exophorics (*Z* = 3.713, *p* < 0.001 and *r* = 0.55) more than orthophorics. Effect size was medium–large (0.30 ≤ *r* < 0.50) for orthophoric subjects and large (*r* > 0.50) for esophorics and exophorics, confirming major changes in absolute distance phoria of each group, which were more pronounced in esophoric and exophoric group compared to orthophoric group.

Stereoacuity without prism was comparable among the three phoria groups (1.67 ± 0.13 log arcsec, equivalent to 46.8″) and showed a small worsening with prism insertion (1.69 ± 0.17 log arcsec, equivalent to 49.4″), without any interaction between prism and phoria (Figure 1). Detailed analysis can be found in the Supplementary Materials (Section S6.2 and Tables S3 and S4).

### 3.2. Posturographic Parameters

**Figure 2:**
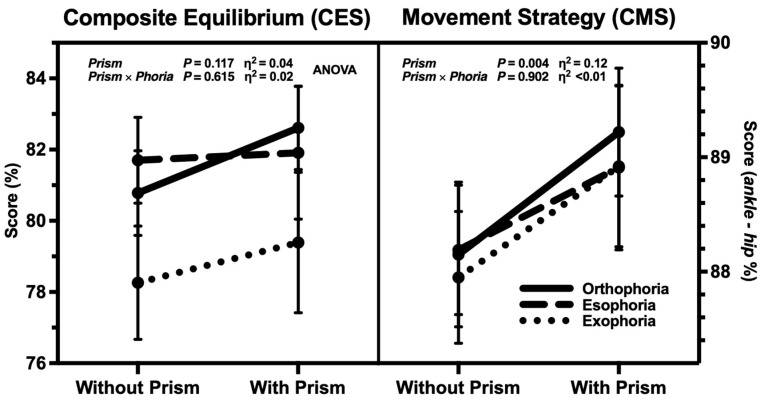
Composite equilibrium (CES) and composite movement strategy scores (CMS) *without* (at baseline) and *with prism*, sorted by *baseline phoria*. A higher CSS denotes a better performance (less sway); a higher CMS indicates a more predominant ankle strategy (versus hip). Dot and bar represent mean and standard error, respectively. Two-way ANOVA statistical significance (*P*) and partial effect size (η^2^) of main effect (*Prism*) and interaction (*Prism × Phoria*) showed only a significant shift of CMS toward ankle strategy for all groups.

**Figure 3:**
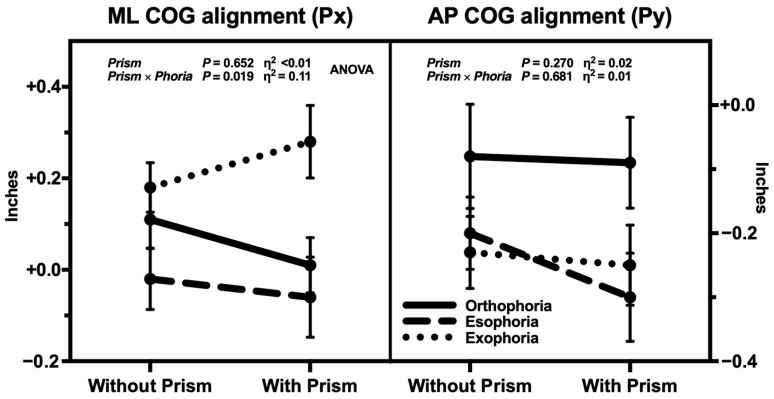
Medio-lateral (ML/Px) and antero-posterior (AP/Py) projection of center of gravity (COG) *without* (at baseline) and *with prism*, sorted by *baseline phoria*. Right and forward displacements are positive, left and backward negative. Dot and bar represent mean and standard error, respectively. Two-way ANOVA statistical significance (*P*) and partial effect size (η^2^) of main effect (*Prism*) and interaction (*Prism × Phoria*) showed no significant displacement of COG, though COG-Px displacement of exophoric subjects was divergent (rightward) from esophoric and orthophoric subjects (leftward).

Posturographic scores (CES and CMS) and center of gravity projection (COG-Px and -Py) without and with prism and grouped by baseline phoria are reported in Table 1 and shown in Figure 2 and Figure 3. Statistical analysis of baseline parameters compared by phoria group (one-way ANOVA) and the main effect of prism and its interaction with baseline phoria (two-way ANOVA) are shown in Table 2.

Baseline horizontal phoria had no statistically significant effect (*p* > 0.05 for all) on any posturographic parameters measured without prism, though COG-Px showed a medium partial effect size (η^2^_p_ = 0.07; Table 2). As emerges from the two-way ANOVA, the main effect of prism on CMS was the only statistically significant result with a medium–large effect size (*p* = 0.004 and η^2^_p_ = 0.12), whereas no statistically significant interaction between prism and baseline phoria was found for any parameters, except for COG-Px (*p* = 0.019) which displayed a medium–large partial effect size (η^2^_p_ = 0.11; Table 2).

Secondary statistical analysis of the main effects of *prism* and *condition* and their interaction with *phoria group* and *ocular dominance* can be found in the Supplementary Materials, Section S6.3 and Table S5. Posturographic scores (CES and CMS) and center of gravity projection (COG-Px and -Py) and pairwise comparisons *without* and *with prism* for each of the six *SOT conditions* are reported in Supplementary Materials, Table S6 and shown in Figures S4 and S5.

In the secondary analysis, CES and CMS were significantly different across all six *conditions* (*p* < 0.001) and this main effect was qualified by a significant interaction between *condition* and *prism* (*p* = 0.020 and *p* < 0.001 for CES and CMS, respectively; Table S5). Pairwise comparisons confirmed that CES *with prism* was significantly higher than CES *without prism* only in the condition SOT 5 (*p* < 0.001) while CMS *with prism* was significantly lower than CMS *without prism* in the condition SOT 1 (*p* = 0.023) and SOT 2 (*p* = 0.018) but significantly higher in SOT 5 (*p* < 0.001; Table S6).

Medio-lateral COG projection (Px) was not significantly different across the six conditions (*p* = 0.716) but showed a significant interaction between *condition* and *prism* (*p* = 0.009), while antero-posterior COG projection (Py) was different across the six conditions (*p* = 0.034) without significant interaction between *condition* and *prism* (*p* = 0.713; Table S5). Pairwise comparisons confirmed that COG-Px *with prism* was marginally lower than COG-Px *without prism* only in the condition SOT 5 (*p* = 0.053; Table S6).

In this second analysis model, none of *ocular dominance*, *phoria* or their combination exhibited any interaction with *prism* or *condition* on any variables (*p* > 0.05), though some had a medium–large partial effect size (Table S5).

## 4. Discussion

Several studies on strabismus suggested a primary role of the visual system in controlling and maintaining postural stability [29,30,31,32,33,34,35,36,37,38]. Both strabismus surgery [39,40,41,42] and prismatic correction [43,44] influence postural control, through changes in binocular vision and stereopsis [5] as well as rearrangements of EOMs pathways [18,19]: according to this hypothesis, EOMs serve also as proprioceptors involved in balance control (postural receptors) [19].

Postural stability has further been related to phoria: subjects presenting vertical heterophoria within the normal range have a worse postural control than subjects with vertical orthophoria when fixating at far but not near distance [10]. Vertical heterophoria influences also the reference posture, thereby affecting head rotation and mobility of the spine and peripheral joints and potentially leading to chronic back pain [24]. Moreover, non-specific chronic low back pain was clinically associated with vertical heterophoria within one-diopter, which improves postural performance if canceled with a prism, suggesting that vertical heterophoria could be the sign of a sensorimotor conflict between vision and proprioceptive inputs leading to altered sensory perception and pain [11].

Previous research has focused mainly on vertical phoria: only a single study explored the effects of horizontal prisms on posture in healthy subjects [25], showing that prism-induced convergence (base in) and vertical vergence without disruption of fusional single vision have a marked negative effect on postural stability compared to prism-induced divergence (base out) and diplopia, which instead do not have any significant influence. However, this study neglected the role of baseline horizontal phoria and considered only the general postural instability. Our study is the first to assess the effects of horizontal prisms and horizontal phoria on posture at the same time, evaluating the influence of baseline phoria (orthophoria, esophoria and exophoria) in conjunction with a low-power convergence-inducing prism (two-diopter, non-dominant eye) in terms of movement strategy (CMS), COG displacement (Px and Py), and global balance performance (CES).

In our study, baseline overall balance performance measured by CES was about 80.3 ± 6.5% (Figure 2 and Table 1), corresponding to a mean COG oscillation of 2.47°, which is consistent with the average score of young healthy subjects [45,46,47,48,49]. Baseline CMS was about 88.1 ± 2.8% (Figure 2 and Table 1), equivalent to a shear force of 2.98 lbs (1.35 kg), implying an ankle-dominant strategy to maintain balance and confirming excellent postural stability, in coherence with published data on young healthy subjects [50,51]. Despite markedly different horizontal phoria among the groups, neither the baseline scores were statistically different; we hypothesize that compensatory mechanisms are likely to intervene under long-standing conditions to maintain consistent balance performance and COG position, in contrast to the wide variability of phoria.

When a two-diopter prism was placed base out on the non-dominant eye, CES remained stable (81.3 ± 8.2%, *p* = 0.259); however, CMS significantly increased to 89.0 ± 3.1% (*p* = 0.004, Figure 2 and Table 1), further shifting toward ankle strategy suggesting better control of postural stability, with an 8% reduction of the shear force to 2.75 lbs (1.25 kg) [3,50]. Although apparently small and sub-clinical, this change reached statistical significance with a medium–large effect size (η^2^_p_ = 0.12). However, the variation of CES was in contrast with the findings of a previous study reporting a marked postural instability following the use of convergence-inducing prisms [25]. This unbalance may be ascribed to the stronger prisms (4- and 8-diopter) than our experiment (2-diopter), besides suggesting a complex effect of prism power.

As emerges from the secondary analysis (Tables S5 and S6), both equilibrium (ES) and movement strategy score (MS) were different across the six SOT conditions and the progression of both was consistent with previous studies [45,46,47,48,49,50,51]. The trial with a prism was comparable to the trial without a prism in all conditions except for SOT 5 (Figure S4), in which both the ES and MS *with a prism* were significantly higher than *without prism* (*p* < 0.001). This suggests that the prism selectively improves balance only in condition 5 with less body sway (higher ES, 70.4 ± 9.7% [3.70° *with prism*] vs. 65.7 ± 11.6% [4.29° *without*]) and more use of ankle strategy (higher MS, 81.6 ± 5.3% [4.59 lbs–2.08 kg *with prism*] vs. 78.2 ± 6.0% [5.46 lbs–2.48 kg *without*]). Condition 5 consists of eye-closed and platform oscillating, is the most challenging SOT trial and mainly relies on the vestibular system to maintain balance [48]. Healthy subjects on stable surfaces primarily move about the ankle joints to correct small deviations, and shift to hip strategy for major corrections when balance becomes more difficult [3,26,50]; for instance, on unstable surfaces (SOT 4), in particular when visual stimulus is absent (SOT 5) or conflicting (SOT 6), as evident from normative values of healthy subjects of different age groups [50,51]. The conditions before SOT 5 rely mainly on other sensory systems to maintain balance: somatic when the platform is fixed and visual when the surroundings are fixed [48]. The conditions are performed consecutively, and the prism is placed and exerts its visual effect during the eyes-open trials before (SOT 1, 3 and 4) [48]. The selective improvement elicited by the prism during an eyes-closed trial seems quite paradoxical. It is possible that this effect persists during the eyes-closed trial immediately after (SOT 5). Apparently, this effect consists of selectively enhancing the vestibular system while unaffecting the somatic and visual systems, and it is overcome by these systems during the other trials, suggesting a complex interaction between all the sensory systems involved in balance control. The selective effect of prism on this condition was apparently unaffected by phoria or ocular dominance, as no statistically significant interaction was found (*p* > 0.05); however, this study was not designed to include ocular dominance or other factors in the analysis and, therefore, no definitive conclusion can be made on the non-significant effects, though suggesting a new perspective on the role of horizontal prisms and phoria on posture.

Baseline COG was projected on average 0.09 ± 0.30 inches (2.3 ± 7.6 mm) to the right of the midline and 0.17 ± 0.32 inches (4.3 ± 8.1 mm) behind the horizontal axis (Figure 3 and Table 1): these findings suggest an antero-posterior projection coherent with previous estimates [52], but a body weight asymmetry skewed toward the right side, which is in contrast with published data [52,53] and it may be explained by the different devices and methods employed. The horizontal displacement of COG was not statistically significant (*p* = 0.652) due to divergent shift of exophoric subjects (rightward) compared to esophoric and orthophoric subjects (leftward) as confirmed by the significant and medium–large effect size of *prism × phoria* interaction (*p* = 0.019 and η^2^_p_ = 0.11), while the antero-posterior projection of COG was stable (*p* = 0.270 and η^2^_p_ = 0.02), without a significant *prism × phoria* interaction (*p* = 0.681 and η^2^_p_ = 0.01). The incoherence between COG-Px and COG-Py may be explained assuming that horizontal phoria has a consistent effect only on medio-lateral displacements but no reproducible effect on antero-posterior shifts due to the intervention of other factors (for instance, EOMs other than medial and lateral recti) [18]. Mechanical stimulation (vibration) of EOMs in standing subjects induces whole body shifts whose direction depends strictly on specific muscles (which is on the horizontal axis for horizontal recti) [18]: it is possible that the divergent displacement of COG projection was related to the changes in the tension of horizontal recti. As the prism always induced a convergent movement increasing the tension of medial recti in exophoric subjects and decreasing it in esophoric subjects, this may justify the different response to the prism between the two groups. Additionally, the secondary analysis suggests that the response of COG to the prism in certain conditions could depend on ocular dominance (i.e., on which eye the prism is placed), as the *condition × prism × dominance* interaction showed a medium–large partial effect size (η^2^_p_ = 0.11 [COG-Px] and η^2^_p_ = 0.08 [COG-Py]; Table S5), although not statistically significant (*p* = 0.225 and *p* = 0.399, respectively). This may further suggest a role of EOMs as proprioceptors influencing posture. However, these hypotheses on the relation between phoria, EOMs tension and posture remain yet to be further explored since this study was not designed to evaluate other arrangements of prisms and did not allow the selective analysis of prism orientation, ocular dominance and laterality. Moreover, COG projection is measured in the preceding half second before each SOT trial [26] and, therefore, small changes in subject positioning across the trials may confound the interpretation of these results, potentially inducing a variability greater than the displacements recorded. CES and CMS are instead based on the difference of sway and shear forces within the same trials and, therefore, are less prone to this interference.

As our primary objective was only to ascertain any effects of horizontal phoria and prisms, our study had some limitations. The study population consisted exclusively of young adults; therefore, these findings may not be applicable to other age groups, particularly older adults or adolescents. Also, all the participants were healthy volunteers and medical students, introducing the potential for self-selection bias and further limiting the generalizability of these results. Future research with a more diverse and representative sample will be necessary. Moreover, our study assessed only the short-term effects of monocular prisms and did not evaluate whether they are long-lasting or otherwise fade over time. These findings should be investigated further by exploring the effects of base-in prisms (inducing esophoria) and stronger prisms to verify whether these changes are reversed by prism orientation and depend on ocular dominance, prism laterality and power.

## 5. Conclusions

Our study confirms that a low-power convergence-inducing prism on the non-dominant eye induces the body to use the ankle joint more independently of individual phoria, suggesting a small improvement in postural control, while maintaining oscillations of the center of gravity unaltered. Prisms seem to enhance the function of the vestibular system selectively, while apparently not affecting the somatic and visual systems, and this effect may be overcome by these systems during other trials. Prismatic lenses could improve balance control and, in selected cases, may be a non-invasive and non-expensive option to manage abnormal posture. Our results support the role of EOMs as proprioceptors involved in balance control (postural receptors).

If the findings of our study are further explored with the use of prisms of higher power over a longer period of time and including a wider cohort of subjects, the monocular prismatic correction could be considered for the adjustment of the anomalous posture of coxo-femoral and scapulo-humeral lines and correction of postural defects (kyphosis, lordosis, etc.) in both adults and children.

## Figures and Tables

**Table 1:** Posturographic parameters.

	Group	Without Prism	With Prism
CES (%) ^a^	Orthophoria (*n* = 23)	80.78 (5.70)	82.61 (5.62)
	Esophoria (*n* = 23)	81.70 (5.78)	81.91 (8.94)
	Exophoria (*n* = 23)	78.26 (7.63)	79.39 (9.46)
	*Pooled* (*N* = 69)	80.25 (6.51)	81.30 (8.18)
CMS (%) ^b^	Orthophoria (*n* = 23)	88.15 (3.03)	89.22 (2.68)
	Esophoria (*n* = 23)	88.19 (2.71)	88.91 (3.45)
	Exophoria (*n* = 23)	87.95 (2.76)	88.92 (3.37)
	*Pooled* (*N* = 69)	88.10 (2.79)	89.02 (3.14)
COG-Px (inch) ^c^	Orthophoria (*n* = 23)	+0.11 (0.30)	+0.01 (0.29)
	Esophoria (*n* = 23)	−0.02 (0.32)	−0.06 (0.42)
	Exophoria (*n* = 23)	+0.18 (0.26)	+0.28 (0.38)
	*Pooled* (*N* = 69)	+0.09 (0.30)	+0.07 (0.39)
COG-Py (inch) ^d^	Orthophoria (*n* = 23)	−0.08 (0.39)	−0.09 (0.34)
	Esophoria (*n* = 23)	−0.20 (0.27)	−0.30 (0.33)
	Exophoria (*n* = 23)	−0.23 (0.27)	−0.25 (0.30)
	*Pooled* (*N* = 69)	−0.17 (0.32)	−0.22 (0.33)

Abbreviations: CES (composite equilibrium score), COG (center of gravity), CMS (composite movement strategy score), Px (medio-lateral projection on *x*-axis), Py (antero-posterior projection on *y*-axis). Note: values are expressed as mean (standard deviation). ^a^ Higher scores are associated with better performances (less sway). ^b^ Higher scores are associated with a more prevalent ankle strategy (versus hip). ^c^ Right displacement of COG is positive, left is negative. ^d^ Forward displacements of COG is positive, backward is negative.

**Table 2:** Statistical analysis.

	Source	Statistic ^a^	Significance ^b^	Effect Size ^c^
CES	Phoria ^d^	1.758	0.180	*0.051*
	Prism ^e^	2.526	0.117	*0.037*
	Prism × Phoria ^f^	0.490	0.615	*0.015*
CMS	Phoria ^d^	0.045	0.956	0.001
	Prism ^e^	8.758	**0.004**	**0.117**
	Prism × Phoria ^f^	0.104	0.902	0.003
COG-Px	Phoria ^d^	2.637	0.079	**0.074**
	Prism ^e^	0.205	0.652	0.003
	Prism × Phoria ^f^	4.212	**0.019**	**0.113**
COG-Py	Phoria ^d^	1.508	0.229	*0.044*
	Prism ^e^	1.238	0.270	*0.018*
	Prism × Phoria ^f^	0.386	0.681	*0.012*

Abbreviations: CES (composite equilibrium score), COG (center of gravity), CMS (composite movement strategy score), Px (medio-lateral projection on *x*-axis), Py (antero-posterior projection on *y*-axis). ^a^ Statistic (*F*): in the one-way ANOVA degrees of freedom (df) were 2 and 66 for *Phoria* (between-groups) and within-groups term, respectively; in the two-way ANOVA df were 1, 2 and 66 for *Prism*, *Prism × Phoria* and error term, respectively. ^b^ Statistical significance (*P*): significant value (*p* < 0.05 in bold). ^c^ Partial eta squared (η^2^_p_): negligible (<0.01, plain text), small–medium (0.01–0.06, in italics), medium–large (0.06–0.14, in bold) and large (>0.14, in bold underlined). ^d^ One-way ANOVA: effect of *Phoria group* at baseline (without prism). ^e^ Two-way ANOVA: main effect of *Prism insertion* (within-subjects factor) irrespective of Phoria group. ^f^ Two-way ANOVA: interaction effect between *Prism* and *Phoria group* (between-subjects factor).

## Data Availability

The datasets used during the current study are available from the corresponding author upon reasonable request.

## Supplementary Materials

The following supporting information can be downloaded at: https://www.mdpi.com/article/10.3390/jcm13247847/s1, Figure S1: Diagram of force plate strain gauges; Figure S2: Center of gravity (COG) projections in quiet stance; Figure S3: Trial frame setup; Figure S4: Equilibrium score (ES) and movement strategy (MS) score *with* and *without prism* across the six sensory organization test (*SOT*) *conditions*; Figure S5: Medio-lateral (ML/Px) and antero-posterior (AP/Py) projection of center of gravity (COG) *without* and *with prism* across the six sensory organization test (*SOT*) *conditions*; Table S1: Sensory organization test (SOT) [48]; Table S2: Subjects characteristics; Table S3: Phoria and stereoacuity; Table S4: Statistical analysis of stereoacuity; Table S5: Secondary analysis of posturographic parameters; Table S6: Pairwise comparisons of secondary analysis; Equations (S1)–(S8).
