# Supplementary material for: Effects on Posture of a Two-Diopter Horizontal Prism Base Out on the Non-Dominant Eye
Source: J Clin Med. 2024 Dec 23;13(24):7847. doi: 10.3390/jcm13247847 (PMC11676230; doi:10.3390/jcm13247847)
Supplement: Supplementary file 1 [file jcm-13-07847-s001.zip › jcm-3320770-supplementary.pdf]

Supplementary Materials

Effects on posture of a two-diopter horizontal prism base out on the non-dominant eye

Davide Marini [1,*,†], Giovanni Rubegni [1,†], Lorenzo Sarti [2], Alessandra Rufa [3], Marco Mandalà [4], Fabio Ferretti [5], Gian Marco Tosi [1] and Mario Fruschelli [1,*]

[1] Ophthalmology Unit, Department of Medicine, Surgery and Neuroscience, University of Siena, Siena, Italy; davide.marini.2@gmail.com (D.M.); giovannirubegni@gmail.com (G.R.); gianmarco.tosi@unisi.it (G.M.T.); mario.fruschelli@unisi.it (M.F.)
[2] School of Orthoptic and Ophthalmologic assistance, Department of Medicine, Surgery and Neuroscience, University of Siena, Siena, Italy; lorenzosartiortottista@gmail.com
[3] Eye Tracking & Visual Application Lab (EVALab), Neurology and Neurometabolic Unit, Department of Medicine, Surgery and Neuroscience, University of Siena, Siena, Italy; alessandra.rufa@unisi.it
[4] Otolaryngology Unit, Department of Medicine, Surgery and Neuroscience, University of Siena, Siena, Italy; mandal@unisi.it
[5] Department of Medicine, Surgery and Neuroscience, University of Siena, Siena, Italy; ferrefa@unisi.it
* Correspondence: davide.marini.2@gmail.com (D.M.); mario.fruschelli@unisi.it (M.F.)
† These authors contributed equally to this work.

S1. Introduction

The EquiTest® NeuroCom® is a stabilometric platform which performs computerized posturography in upright stance in both static and dynamic conditions [26]. Sensory organization test (SOT) is one of the standardized protocols of the platform, which evaluates the interaction between the somatosensory, visual and vestibular systems contributing to postural control, under six different sensorial conditions. The balance control is assessed in terms of body sway (*equilibrium score*, ES), the relative torques of hip and ankle joints used to maintain balance (*movement strategy score*, MS), and displacements of the *center of gravity projection* on the support surface (COG-Px and -Py, respectively) [48].

S2. Mechanics

The subject is placed on a dual force plate and surrounded by a panorama reproducing an open space, both oscillating in exact synchrony with each other, wearing a safety harness [26].

The force plate consists of two 9 × 18 inches footplates connected by a pin joint. The footplates are sustained by four strain gauges mounted on a supporting center plate and positioned 4.00 inches from the y-axis and 4.20 inches from the x-axis either side, and a fifth transducer attached to the center plate directly beneath the pin joint (Figure S1). The four strain gauges measure the four vertical forces applied to the force plate: right-front (RF), right-rear (RR), left-front (LF) and left-rear (LR); while the center strain gauge measures the shear force along the y-axis (Fh) [26].

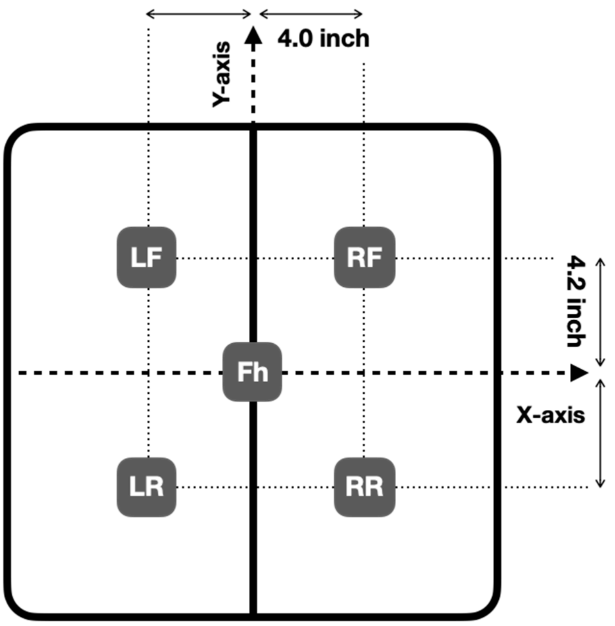

Figure S1. Diagram of force plate strain gauges. The four transducers inside the support measure vertical forces: LF (left front), RF (right front), LR (left rear), RR (right rear). The single transducer at the center measures horizontal shear force (Fh).

S3. Examination

The examination is composed of a triplet of 20-seconds trials for each of the six conditions changing for support plate inputs (fixed and sway-referenced) and visual inputs (fixed, eyes closed and sway-referenced), which are summarized in Table S1 [48].

Table S1. Sensory organization test (SOT) [48].

Condition	Eyes	Surroundings [a]	Platform [b]	Sensory system [c]
**1**	Open	Fixed	Fixed	Somatic
**2**	Closed	NA	Fixed	Somatic
**3**	Open	Sway referenced	Fixed	Somatic
**4**	Open	Fixed	Sway referenced	Visual
**5**	Closed	NA	Sway referenced	Vestibular
**6**	Open	Sway referenced	Sway referenced	Vestibular

Abbreviations: NA, not applicable.
[a] Surrounding may be fixed, oscillate alone or in conjunction with platform.
[b] Platform may be fixed or oscillate.
[c] It denotes on which sensory system subjects rely to maintain balance in case of sensory conflict.

S4. Calculations

4.1. Vertical and horizontal forces

The system calculates two different forces and two geometric points by which the equilibrium performance and movement strategy scores are then estimated. The *total vertical force* (Fv) is the sum of vertical forces exerted each transducer at the four corners:

$$Fv = RF + RR + LF + LR, \tag{S1}$$

where RF, RR, LF and LR are the four vertical forces, respectively (Figure S1). The *total horizontal force* (Fh) represents the shear force (SH) and is measured directly by the central transducer:

$$Fh = SH. \tag{S2}$$

The *medio-lateral center of vertical force* (Px) is the projection of the COG on the *x*-axis:

$$Px = \frac{RF + RR - LF - LR}{Fv} \cdot 4.00\ (inch), \tag{S3}$$

where right displacements are positive and left negative. The *antero-posterior center of vertical force* (Py) represents the projection of the COG on the *y*-axis:

$$Py = \frac{RF + LF - RR - LR}{Fv} \cdot 4.20\ (inch), \tag{S4}$$

where forward displacements are positive, and backward negative [26].

4.2. Center of gravity (COG)

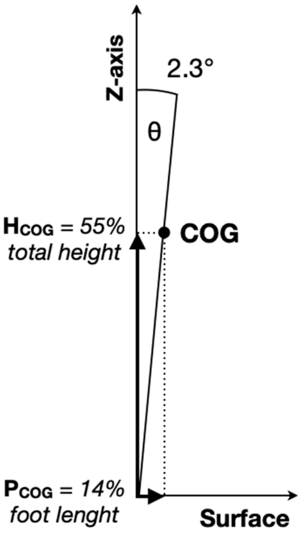

Figure S2. Center of gravity (COG) projections in quiet stance. H_{COG} (COG height), P_{COG} (COG projection on support surface).

In quiet upright stance, COG is assumed at a height (H_{COG}) 55% of the subject total height and 14% of the foot length (P_{COG}) in front of the medial malleolus bone in the ankle joint (Figure S2). Therefore, COG is positioned at a tilted angle of 2.3° forward from the vertical line passing through the ankle joints, and it is projected on a point (center of foot support) slightly forward of the ankle joint between front and rear foot edges and halfway feet lateral edges. Initial COG alignment is determined as the average antero-posterior COG position in the preceding half second before each SOT trial. The *antero-posterior COG sway angle* (θ) is calculated as follows:

$$\theta = arcsin\left(\frac{P_{COG}}{H_{COG}}\right) - 2.3°. \tag{S5}$$

S4.3. Equilibrium score and movement strategy

The *equilibrium score* (ES) quantifies the maximum range of antero-posterior oscillation (COG sway) on each trial:

$$ES = \left(1 - \frac{\theta_{max} - \theta_{min}}{12.5°}\right) \cdot 100\ (\%), \tag{S6}$$

where 12.5° is the theoretical upper limit of normal antero-posterior sway. The score ranges from 0 to 100: in case of fall or negative value the score is assumed zero, while higher scores are associated to smaller area of oscillation. The *composite equilibrium score* (CES) measures the overall level of performance and is the weighted average of all sensory conditions scores, where conditions 3 to 6 are given three times the weight of conditions 1 and 2 [26]:

$$CES = \frac{ES_1 + ES_2 + 3 \cdot (ES_3 + ES_4 + ES_5 + ES_6)}{14}. \quad \text{(S7)}$$

The *movement strategy score* (MS) quantifies the ankle and hip movements used to maintain balance:

$$MS = \left(1 - \frac{SH_{max} - SH_{min}}{25 \text{ lbs}}\right) \cdot 100\ (\%), \quad \text{(S8)}$$

where 25 lbs (11.3 kg) is the theoretical difference between the maximum (SH_{max}) and lowest shear force (SH_{min}) generated by a test group of normal subjects using only hip torque to balance. Higher scores are associated to a more use of ankle than hip strategy. Hip movements generate horizontal shear forces against the support surface proportional to the second time derivative of hip joint angle (angular acceleration), while vertical forces change only if hip movements determine a variation of COG sway angle [26]. The software directly provides a global score for equilibrium (composite equilibrium score), which is the weighted average across the six conditions; however, it does not return a composite score for movement strategy. Therefore, we assigned a "composite movement strategy score" (CMS) with the same procedure and weights:

$$CMS = \frac{MS_1 + MS_2 + 3 \cdot (MS_3 + MS_4 + MS_5 + MS_6)}{14}. \quad \text{(S9)}$$

This method of computing CES and CMS gives importance to the most challenging conditions in which either the platform or the surroundings oscillates [26]. The same was done for mediolateral (Px) and antero-posterior (Py) COG projection.

S5. Trial frame

The setup of the two-diopter prism is explained in Figure S3.

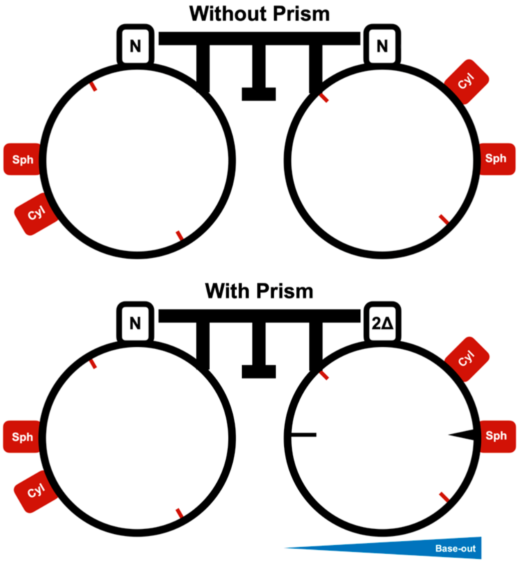

Figure S3. This is an example of trial frame setup in a case of right-eye dominance. To sham the presence of the prism, in the experiment *without prism* a neutral lens (N) was placed before both eyes with the proper spherical (Sph) and cylindrical (Cyl) refraction, while in the experiment *with prism* the two-diopter prism is placed base out before the non-dominant left eye (2 Δ) while a neutral lens before the right eye (N). The best effort was made to prevent subjects from knowing the trial frame composition.

S6. Statistical analysis

S6.1. Subjects characteristics

The characteristics of subjects are reported in Table S2. All the items of the self-reported questionnaire (headache, asthenopia, dizziness and pain) were comparable among the three phoria groups. The exophoria group showed a slight prevalence of female subjects, frontal headache, eye redness and eye strain, which were all below statistical significance.

Table S2. Subjects characteristics.

		Group			Significance [a]	Effect size [b]
		Orthophoria (n=23)	Esophoria (n=23)	Exophoria (n=23)		
**Gender (F/M)**		13/10 (56%)	15/8 (65%)	19/4 (83%)	0.199	**0.233**
**Dominance (R/L)**		19/4 (83%)	14/9 (61%)	16/7 (70%)	0.304	0.197
**Headache (Y/N)**	*Frontal*	9/14 (39%)	4/19 (17%)	8/15 (35%)	0.257	**0.204**
	Temporal	5/18 (22%)	2/21 (9%)	6/17 (26%)	0.394	0.189
	Occipital	1/22 (4%)	1/22 (4%)	0/23 (0%)	>0.999	0.122
**Asthenopia (Y/N)**	*Blurring*	5/18 (22%)	5/18 (22%)	4/19 (17%)	>0.999	0.051
	Redness	4/19 (17%)	5/18 (22%)	10/13 (44%)	0.124	**0.255**
	Diplopia	1/22 (4%)	3/20 (13%)	3/20 (13%)	0.685	0.136
	Tonic accommodation	11/12 (48%)	11/12 (48%)	9/14 (39%)	0.867	0.082
	Eye strain	3/20 (13%)	3/20 (13%)	8/15 (35%)	0.138	**0.255**
	Reading fatigue	10/13 (44%)	9/14 (39%)	9/14 (39%)	>0.999	0.042
**Dizziness (Y/N)**		1/22 (4%)	3/20 (13%)	3/20 (13%)	0.684	0.136
**Pain (Y/N)**	*Neck*	9/14 (39%)	8/15 (35%)	8/15 (35%)	>0.999	0.043
	Shoulders	6/17 (26%)	7/16 (30%)	7/16 (30%)	>0.999	0.045
	Low-back	6/17 (26%)	7/16 (30%)	8/15 (35%)	0.945	0.077

Abbreviations: F, female; L, left eye; M, male; N, no; R, right eye; Y, yes. [a] Statistical significance (P): Fisher's exact test, significant values ($p < 0.05$) in bold. [b] Cramer's *V*: negligible (<0.20, plain text), moderate (0.20-0.50, in bold), strong (>0.50, in bold underlined).

S6.2. Phoria and stereoacuity

Distance phoria and stereoacuity are reported in Table S3. Statistical analysis of distance phoria is reported in Section 3.1 of manuscript, and stereoacuity is reported in Table S4. Stereoacuity without prism was comparable among the three phoria groups (1.67 ± 0.13 log arcsec, equivalent to 46.8") and showed a small worsening with prism insertion (1.69 ± 0.17 log arcsec, equivalent to 49.4"), without any interaction between prism and phoria.

Table S3. Phoria and stereoacuity.

	Group	Without Prism	With Prism
**Phoria (Δ)** [a]	Orthophoria (n=23)	+0.50 (+0.50, +0.50)	+1.00 (+0.50, +2.00)
	Esophoria (n=23)	−3.00 (−7.00, −2.00)	−2.00 (−4.50, +0.00)
	Exophoria (n=23)	+2.00 (+1.00, +6.00)	+4.00 (+2.00, +7.50)
**Stereoacuity (log arcsec)** [b]	Orthophoria (n=23)	1.64 (0.08) eq. 44.0"	1.66 (0.13) eq. 46.1"
	Esophoria (n=23)	1.65 (0.12) eq. 44.9"	1.72 (0.20) eq. 52.4"
	Exophoria (n=23)	1.71 (0.16) eq. 51.7"	1.76 (0.22) eq. 56.8"
	Pooled (N=69)	1.67 (0.13) eq. 46.8"	1.69 (0.17) eq. 49.4"

Abbreviations: Δ (prismatic diopter), log arcsec (base-10 logarithm of stereoacuity seconds of arc). Note: normally distributed variable is expressed as mean (standard deviation) and non-normally

distributed as median (interquartile range). [a] Distance phoria: exophoria is positive, esophoria is negative, and orthophoria is defined as +0.50 Δ. [b] Higher values are associated to worse stereopsis.

Table S4. Statistical analysis of stereoacuity.

	Source	Statistic [a]	Significance [b]	Effect size [c]
**Stereoacuity**	Phoria [d]	2.220	0.117	**0.063**
	Prism [e]	5.026	**0.028**	**0.071**
	Prism × Phoria [f]	1.418	0.249	*0.041*

[a] Statistic (F): in the one-way ANOVA degrees of freedom (df) were 2 and 66 for *Phoria* (between-groups) and within-groups term, respectively; in the two-way ANOVA df were 1, 2 and 66 for *Prism*, *Prism* × *Phoria* and error term, respectively. [b] Statistical significance (P): significant value ($p < 0.05$ in bold). [c] Partial eta squared (η^2_P): negligible (<0.01, plain text), small-medium (0.01-0.06, in italics), medium-large (0.06-0.14, in bold) and large (>0.14, in bold underlined). [d] One-way ANOVA: effect of *Phoria group* at baseline (without prism). [e] Two-way ANOVA: main effect of *Prism insertion* (within-subjects factor) irrespective of Phoria group. [f] Two-way ANOVA: interaction effect between *Prism* and *Phoria group* (between-subjects factor).

S6.3. Secondary analysis

A secondary analysis was done to determine whether the effect of prism on posturographic scores depended on which eye was placed (*ocular dominance*) and to verify whether the effect of prism was different only in certain *SOT conditions* (Table S5). Posturographic scores (CES and CMS) and center of gravity projection (COG-Px and -Py) without and with prism for each of the six conditions are reported in Table S6 and showed in Figure S4 and S5. CES and CMS were significantly different across all the six conditions (P<0.001) and the main effect of *condition* on CES and CMS was qualified by a significant interaction between *condition* and *prism* (P=0.020 and P<0.001, respectively). Pairwise comparisons (Table S6) confirmed that CES *with prism* was significantly higher than CES *without prism* only in the condition SOT 5 (p<0.001) while CMS with prism was significantly lower than CMS *without prism* in the condition SOT 1 (p=0.023) and SOT 2 (p=0.018) but significantly higher in SOT 5 (p<0.001).

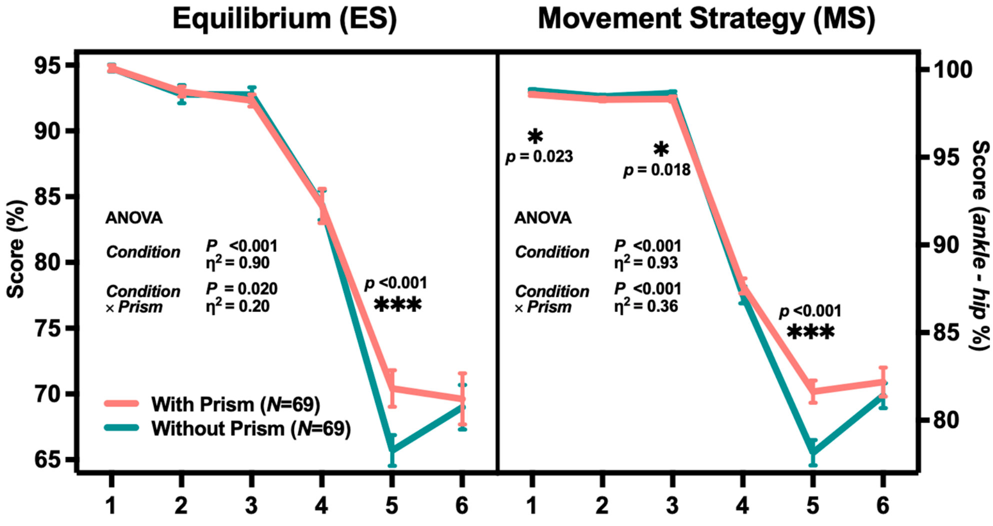

Figure S4. Equilibrium score (ES) and movement strategy (MS) score *without* and *with prism* across the six sensory organization test (SOT) *conditions*. A higher ES denotes a better performance (less sway); a higher MS indicates a more predominant ankle strategy (versus hip). Line and bar represent mean and standard error, respectively. Repeated measures ANOVA statistical significance (P) and partial effect size (η^2) of main effect (*Condition*) and interaction (*Condition* × *Prism*) showed a significant main effect and interaction on both ES and MS. Pairwise comparisons confirmed a significantly better performance (ES) *with prism* than *without prism* only in condition SOT 5, a lower use of ankle strategy (MS) in conditions SOT 1 and SOT 2, while a significantly higher use of ankle strategy in SOT 5 (* p<0.05, ** p<0.01, *** p<0.001).

Center of gravity projection (COG-Px) was not significantly different across the six conditions (P=0.716) but showed a significant interaction between *condition* and *prism* (P=0.009), while COG-Py was different across the six conditions (P=0.034) without a significant interaction between *condition* and *prism* (P=0.713). Pairwise comparisons (Table S6) confirmed that COG-Px *with prism* was marginally lower than COG-Px *without prism* only in the condition SOT 5 (p=0.053).

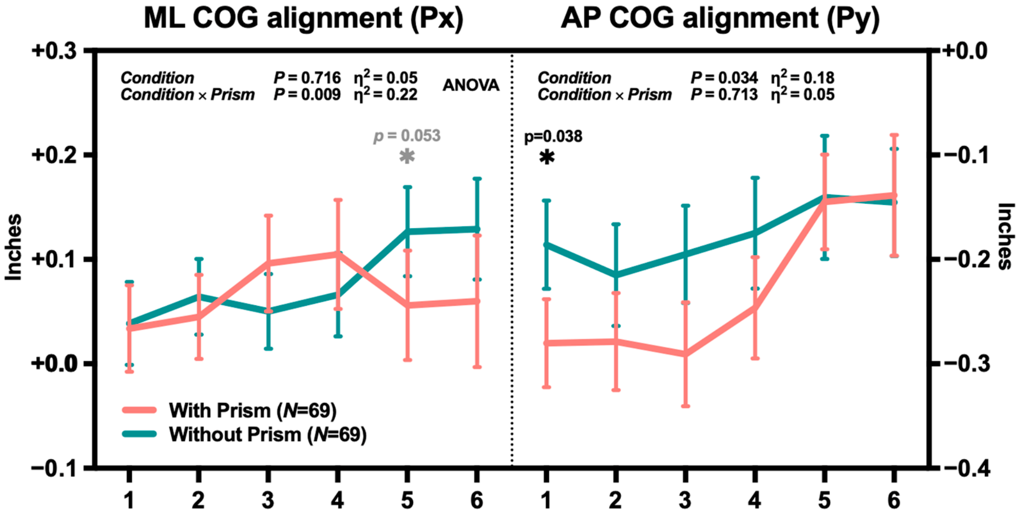

Figure S5. Medio-lateral (ML/Px) and antero-posterior (AP/Py) projection of center of gravity (COG) *without* and *with prism* across the six sensory organization test (*SOT*) *conditions*. Right and forward displacements are positive, left and backward negative. Line and bar represent mean and standard error, respectively. Repeated measures ANOVA statistical significance (*P*) and partial effect size (η^2) of main effect (*Condition*) and interaction (*Condition* × *Prism*) showed a significant main effect without interaction on COG-Px and a significant interaction without main effect on COG-Py. Pairwise comparisons confirmed a marginal leftward displacement of COG *with prism* insertion only in condition SOT 5 and a significant backward displacement of COG in SOT 1 (* p<0.05, ** p<0.01, *** p<0.001).

In this second analysis model, none of *ocular dominance*, *phoria* or their combination exhibited any interaction with *prism* or *condition* on any variables (P>0.05), though some had a medium-large effect size (Table S5).

Table S5. Secondary analysis of posturographic parameters.

	Effect	Statistic [a]	Significance [b]	Effect size [c]
CES	*Prism* [d]	1.598	0.212	*0.025*
	Prism × *Phoria* [e]	0.647	0.527	*0.020*
	Prism × *Dominance* [d]	0.211	0.647	0.003
	Prism × *Dominance* × *Phoria* [e]	1.290	0.283	*0.039*
	Condition [f]	110.1	**<0.001**	**<u>0.903</u>**
	Condition × *Phoria* [g]	0.708	0.715	*0.057*
	Condition × *Dominance* [f]	0.970	0.444	**0.076**
	Condition × *Dominance* × *Phoria* [g]	1.252	0.266	**0.096**
	Condition × *Prism* [f]	2.937	**0.020**	**<u>0.199</u>**
	Condition × *Prism* × *Phoria* [g]	0.463	0.911	*0.038*
	Condition × *Prism* × *Dominance* [f]	0.658	0.657	*0.053*
	Condition × *Prism* × *Dominance* × *Phoria* [g]	0.841	0.590	**0.067**
CMS	*Prism* [d]	5.258	**0.025**	**0.077**
	Prism × *Phoria* [e]	0.031	0.970	0.001
	Prism × *Dominance* [d]	0.209	0.649	0.003
	Prism × *Dominance* × *Phoria* [e]	0.313	0.733	*0.010*

	Condition [f]	152.6	**<0.001**	**0.928**
	Condition × Phoria [g]	0.283	0.984	*0.023*
	Condition × Dominance [f]	0.733	0.601	*0.059*
	Condition × Dominance × Phoria [g]	1.584	0.119	**0.118**
	Condition × Prism [f]	6.542	**<0.001**	**0.357**
	Condition × Prism × Phoria [g]	0.547	0.853	*0.044*
	Condition × Prism × Dominance [f]	0.377	0.862	*0.031*
	Condition × Prism × Dominance × Phoria [g]	0.606	0.806	*0.049*
COG-Px	*Prism* [d]	0.042	0.838	0.001
	Prism × Phoria [e]	3.151	**0.050**	**0.091**
	Prism × Dominance [d]	2.105	0.152	*0.032*
	Prism × Dominance × Phoria [e]	0.007	0.993	<0.001
	Condition [f]	0.579	0.716	*0.047*
	Condition × Phoria [g]	1.010	0.439	**0.079**
	Condition × Dominance [f]	1.331	0.264	**0.101**
	Condition × Dominance × Phoria [g]	1.098	0.370	**0.085**
	Condition × Prism [f]	3.404	**0.009**	**0.224**
	Condition × Prism × Phoria [g]	0.557	0.846	*0.045*
	Condition × Prism × Dominance [f]	1.437	0.225	**0.109**
	Condition × Prism × Dominance × Phoria [g]	0.599	0.812	*0.048*
COG-Py	*Prism* [d]	1.213	0.275	*0.019*
	Prism × Phoria [e]	0.327	0.722	*0.010*
	Prism × Dominance [d]	0.309	0.581	0.005
	Prism × Dominance × Phoria [e]	1.831	0.169	*0.055*
	Condition [f]	2.599	**0.034**	**0.181**
	Condition × Phoria [g]	1.057	0.401	**0.082**
	Condition × Dominance [f]	0.584	0.712	*0.047*
	Condition × Dominance × Phoria [g]	1.138	0.340	**0.088**
	Condition × Prism [f]	0.582	0.713	*0.047*
	Condition × Prism × Phoria [g]	1.138	0.340	**0.088**
	Condition × Prism × Dominance [f]	1.046	0.399	**0.081**
	Condition × Prism × Dominance × Phoria [g]	0.333	0.970	*0.027*

Abbreviations: CES (composite equilibrium score), COG (center of gravity), CMS (movement strategy score), Px (medio-lateral projection on *x*-axis), Py (antero-posterior projection on *y*-axis). [a] Statistic (*F*): multivariate test (Wilks' Lambda exact statistics) of repeated measures ANOVA with *SOT condition* and *Prism insertion* as within-subjects factors and *Ocular dominance* and *Phoria group* as between-subjects factors. [b] Statistical significance (P): significant value (P<0.05 in bold). [c] Partial eta squared (η^2_P): negligible (<0.01, plain text), small-medium (0.01-0.06, in italics), medium-large (0.06-0.14, in bold) and large (>0.14, in bold underlined). [d] Degrees of freedom (df) of hypothesis and error were 1 and 63, respectively. [e] Degrees of freedom (df) of hypothesis and error were 2 and 63, respectively. [f] Degrees of freedom (df) of hypothesis and error were 5 and 59, respectively. [g] Degrees of freedom (df) of hypothesis and error were 10 and 118, respectively.

Table S6. Pairwise comparisons of secondary analysis.

	**Condition** [a]	**Without Prism**	**With Prism**	**Significance** [b]
Equilibrium Score (%) [c]	SOT 1 (*N*=69)	94.78 (2.23)	94.78 (1.84)	>0.999
	SOT 2 (*N*=69)	92.79 (5.68)	92.99 (3.06)	0.637
	SOT 3 (*N*=69)	92.75 (4.62)	92.31 (3.82)	0.151
	SOT 4 (*N*=69)	84.34 (9.32)	84.30 (10.9)	0.957
	SOT 5 (*N*=69)	65.70 (9.68)	70.42 (11.6)	**<0.001**
	SOT 6 (*N*=69)	69.00 (14.1)	69.62 (16.1)	0.672
	SOT 1 (*N*=69)	98.81 (0.68)	98.58 (0.70)	**0.023**

	SOT 2 (*N*=69)	98.47 (0.81)	98.29 (0.88)	0.073
Movement Strategy Score (%) [d]	SOT 3 (*N*=69)	98.65 (0.92)	98.32 (1.21)	**0.018**
	SOT 4 (*N*=69)	87.15 (3.99)	87.66 (3.55)	0.284
	SOT 5 (*N*=69)	78.15 (6.01)	81.63 (5.29)	**<u><0.001</u>**
	SOT 6 (*N*=69)	81.41 (5.92)	82.18 (6.85)	0.361
COG-Px (inch) [e]	SOT 1 (*N*=69)	+0.04 (0.33)	+0.03 (0.34)	0.903
	SOT 2 (*N*=69)	+0.06 (0.30)	+0.04 (0.33)	0.597
	SOT 3 (*N*=69)	+0.05 (0.30)	+0.10 (0.38)	0.268
	SOT 4 (*N*=69)	+0.07 (0.33)	+0.10 (0.43)	0.304
	SOT 5 (*N*=69)	+0.13 (0.35)	+0.06 (0.44)	*0.053*
	SOT 6 (*N*=69)	+0.13 (0.40)	+0.06 (0.52)	0.139
COG-Py (inch) [f]	SOT 1 (*N*=69)	−0.19 (0.35)	−0.28 (0.35)	**0.038**
	SOT 2 (*N*=69)	−0.21 (0.40)	−0.28 (0.39)	0.276
	SOT 3 (*N*=69)	−0.20 (0.39)	−0.29 (0.41)	0.105
	SOT 4 (*N*=69)	−0.17 (0.44)	−0.25 (0.40)	0.253
	SOT 5 (*N*=69)	−0.14 (0.49)	−0.14 (0.38)	0.946
	SOT 6 (*N*=69)	−0.15 (0.43)	−0.14 (0.48)	0.923

Abbreviations: CES (composite equilibrium score), COG (center of gravity), CMS (movement strategy score), Px (medio-lateral projection on *x*-axis), Py (antero-posterior projection on *y*-axis), SOT (sensory organization test). Note: values are expressed as mean (standard deviation). [a] Condition of sensory organization test (SOT). [b] Pairwise comparisons between *Without* and *With Prism* for each condition. [c] Higher scores are associated to better performances (less sway). [d] Higher scores are associated to a more prevalent ankle strategy (versus hip). [e] Right displacement of COG is positive, left is negative. [f] Forward displacements of COG is positive, backward is negative.

References (from the manuscript)

26. NeuroCom® International Inc. *Instruction for use: EquiTest® system operator's manual*; version 8; NeuroCom® International Inc: Clackamas, OR, USA, 2003; pp. 1–9.
48. Natus® Medical Inc. *Balance manager clinical operation guide*; Natus® Medical Inc: Seattle, WA, USA, 2014.
